# Acute effects of resistance-type and cycling-type high-intensity interval training on arterial stiffness, cardiac autonomic modulation and cardiac biomarkers

**DOI:** 10.1186/s13102-024-00806-8

**Published:** 2024-01-11

**Authors:** Tianjiao Wang, Jun Mao, Shumin Bo, Li Zhang, Qing Li

**Affiliations:** College of Kinesiology and Health, Capital University of Physical Education and Sports, Beijing, China

**Keywords:** Resistance-type high-intensity interval training, Cycling-type high-intensity interval training, Cardiovascular

## Abstract

**Background:**

High-intensity interval training (HIIT) has been shown to enhance cardiovascular health. However, there is a lack of research investigating the specific cardiovascular effects of different HIIT training modes. Therefore, this study aimed to compare the acute effects of cycling-type high intensity interval training (C-HIIT) and resistance-type high intensity interval training (R-HIIT) on arterial stiffness, cardiac autonomic modulation, and cardiac biomarkers in healthy young men.

**Methods:**

This is a cross-over randomized trial. Eleven healthy active young men took part in both C-HIIT and R-HIIT. Cardio-ankle vascular index (CAVI), heart rate variability (HRV), and systolic blood pressure (SBP) were measured before, immediately and 30 min after the exercise in C-HIIT and R-HIIT. Meanwhile, blood samples for cardiac troponin-T (cTnT) and amino-terminal pro-B-type natriuretic peptide (NT-proBNP) were assessed using ELISA before, 5min and 35min after exercise.

**Results:**

There was a significant time × group interaction effect (*P* = 0.019, *η*_*p*_^*2*^ = 0.182) and time main effect for ⊿CAVI (*P* < 0.001, *η*_*p*_^*2*^ = 0.729), and R-HIIT resulted in a more significant reduction in ⊿CAVI compared to C-HIIT (− 0.60 ± 0.30, *P* = 0.043, *d* = 0.924) immediately after exercise. There was a significant time main effect was observed for SBP (*P* = 0.001, *η*_*p*_^*2*^ = 0.304). A significant time main effect for lnHF (*P* < 0.001, *η*_*p*_^*2*^ = 0.782), lnRMSSD (*P* < 0.001, *η*_*p*_^*2*^ = 0.693), and LF/HF (*P* = 0.001, *η*_*p*_^*2*^ = 0.302) of HRV was observed. A significant time main effect was observed for cTnT (*P* = 0.023, *η*_*p*_^*2*^ = 0.193) and NT-proBNP (*P* = 0.001, *η*_*p*_^*2*^ = 0.334) of cardiac biomarkers.

**Conclusion:**

R-HIIT and C-HIIT elicited similar acute responses in cardiac autonomic modulation and cardiac biomarkers. However, R-HIIT was more effective in reducing arterial stiffness in healthy young men. Furthermore, the increase in cardiac biomarkers induced by both C-HIIT and R-HIIT was reversible.

**Trial registration:**

The study was prospectively registered on 22 February 2022 at www.chictr.org.cn with identification number ChiCTR2200056897.

**Supplementary Information:**

The online version contains supplementary material available at 10.1186/s13102-024-00806-8.

## Introduction

High-intensity interval training (HIIT) involves alternating short bursts of high-intensity exercise, typically reaching 85% of peak heart rate (HR_peak_), with recovery periods of rest or light exercise at around 70% of HR_peak_ [1]. The effectiveness of HIIT can be influenced by various factors, such as the duration of work and rest, work-rest ratio, recovery methods, and the mode of training etc. [2–4]. Traditional HIIT, typically performed on a cycle ergometer or treadmills, can improve cardiorespiratory fitness in a short period [5, 6]. On the other hand, resistance-type high intensity interval training (R-HIIT), which involves free weights (barbells, dumbbells, or kettlebells), specialized equipment, and body weight, has gained attention in recent years [7]. Sheykhlouvand reported that 8 weeks of using one-armed cable row R-HIIT for kayak sprint athletes increased both cardiorespiratory fitness and muscle strength, and McRae showed that 22 active women improved cardiorespiratory endurance as well as muscle mass after 4 weeks of whole-body R-HIIT [8, 9]. Accordingly, it is important to note that R-HIIT offers greater exercise benefits than cycling-type high intensity interval training (C-HIIT).

Many studies have investigated the acute effects of exercise on arterial stiffness in humans [10, 11]. For example, Wang reported that the acute effect of interval training on improved arterial stiffness lasts longer than continuous exercise when exercise intensity and duration are equivalent [12]. On the other hand, previous studies have reported that both the number of exercise bouts and the duration of intervals can influence the impact of interval training on arterial stiffness [13, 14]. Therefore, it can be concluded that acute interval training appears to be the most effective method for improving arterial stiffness. However, there is still uncertainty regarding the different interval training modes and their effects on arterial stiffness.

Heart rate variability (HRV) is a non-invasive method that is used to assess the cardiac autonomic reactivity, which shows the regulation of heart rate by the parasympathetic and sympathetic systems [15]. It is worth noting that acute exercise can temporarily disrupt HRV [3, 16]. Schaun reported similar effects on HRV between whole-body aerobics HIIT and C-HIIT, although the exercise intensity was not matched in their study [3]. To address this limitation, our study will use the rating of perceived exertion (RPE) method to ensure that the intensity of the two HIIT modes is matched. This method has been successfully used by Rønnestad and Järvinen in previous studies [17, 18].

Cardiac troponin-T (cTnT) is associated with elevated cardiac injury, has been shown to increase after prolonged and high-intensity exercise among healthy individuals [19]. Similarly, amino-terminal pro-B-type natriuretic peptide (NT-proBNP) is also a useful cardiac biomarker for diagnosing myocardial injury and prognosis [20]. Elevated NT-proBNP reflects increased myocardial wall stress [20]. Most studies have focused on the effects of cTnT and NT-proBNP during endurance training or competition [21]. However, it’s uncertain what the acute effects of different modes of HIIT are on cardiac biomarkers.

Therefore, the purpose of this study was to compare the acute effects of C-HIIT and R-HIIT on arterial stiffness, cardiac autonomic modulation, and cardiac biomarkers with the same duration and intensity, but performed with different training modes. We hypothesized that the effects of C-HIIT and R-HIIT on arterial stiffness, cardiac autonomic modulation, and cardiac biomarkers would not differ.

## Material and methods

### Participants

The 11 subjects in this study were all undergraduate students majoring in Physical Education from the Capital Institute of Physical Education and Sports in Beijing, China. Their mean age was 21.36 ± 2.46 years, height was 179 ± 5 cm, weight was 72. 8 ± 10.7 kg, percentage of body fat was 18.0 ± 6.7%, and body mass index was 23 ± 3 kg/m^2^ (Table 1). The inclusion criteria for participants were as follows: (1) They had to be within the age range of 18–28 years old and engage in at least two systematic training sessions per week. Participants were prohibited from using any additional dietary supplementation. (2) They were not allowed to consume androgens or other performance-enhancing drugs. Before the start of the experiment, all subjects were familiar with the experimental procedure and were required to sign an informed consent form. The minimal sample size of 12 (six in each group) was determined by a priori analyses using G*Power software (version 3.1.9.2) based on the following parameters: an alpha level of 0.05, a power (1-beta) equal to 0.8, and an effect size of 0.4.

**Table 1:** Anthropometric and physiological data for participants (*n* = 11)

Parameter	Mean ± SD
Age (years)	21.36 ± 2.46
Height (cm)	179 ± 5
Weight (kg)	72.8 ± 10.7
Body fat percentage (%)	18.0 ± 6.8
Body mass index (kg/m^2^)	23 ± 3
V̇O_2max_ (ml/kg/min)	46.06 ± 9.29
HR_max_ (bpm)	197 ± 19

### Experimental design

A randomized crossover design was used in this study. Each participant performed three experiments. First, participants performed anthropometric measurements maximal incremental cycling test to determine maximal oxygen consumption (V̇O_2_), maximal heart rate (HR_max_), and peak output power (PPO). They were then familiarized with the experimental procedures and squatting movement standards.

**Fig. 1:**
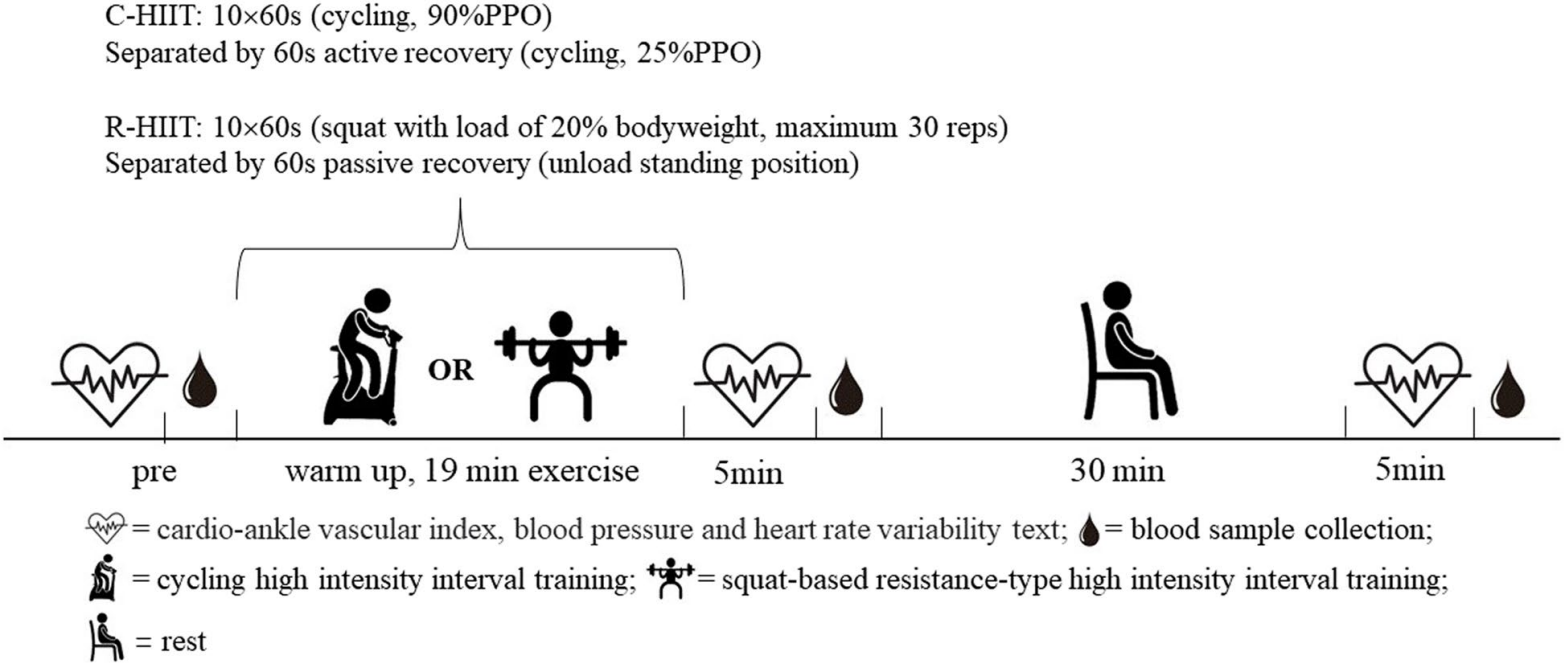
Exercise protocol of the present study C-HIIT: cycling-type high intensity interval training; R-HIIT: resistance-type high intensity interval training

To assess arterial stiffness, we utilized the cardio-ankle vascular index (CAVI), which is an index that measures the stiffness of the arterial tree from the aorta to the ankle [22]. Participants randomly performed C-HIIT and R-HIIT respectively in the second or third experiments and were measured CAVI, systolic blood pressure (SBP), HRV and collected blood samples at previous, immediately, and 30 min after each exercise bout (Fig. 1). All visit sessions are separated by at least a week washout period between two HIIT sessions. Both C-HIIT and R-HIIT consisted of a 10-minute warm-up, 19 minutes of exercise intervention, and 30 minutes of sitting after exercise. All participants abstained from vigorous activity and from alcohol or caffeine intake over the day before the protocols. Participants completed the tests and exercise sessions from 9:00 a.m. to 12:00 a.m. of the day, and measurements were performed in a temperature-controlled (22–24 °C) and humidity-controlled (40–50%) room. Ethical approval was granted by the Capital University of Physical Education and Sports Ethical Committee. This study adhered to CONSORT guidelines for randomized controlled trials and had been registered on Chinese Clinical Trial Registry (www.chictr.org.cn, registration number: ChiCTR2200056897).

We used a HIIT protocol (10 × 60s high-intensity intervals separated by 60s of active recovery), which has been shown to be efficacious and practical in healthy young men [23]. During the second and third visits to the laboratory, participants were randomly assigned to one of the two modes of exercise: C-HIIT and R-HIIT **(**Fig. 1**)**. Throughout the test, subjects received verbal encouragement. Our pilot study revealed that participants could perform two modes of HIIT with similar RPE. Therefore, the intensity of R-HIIT and C-HIIT was considered to be the same. This method has been used by other researchers [17, 18].

Participants in this study underwent a 10-minute warm-up session before engaging in the C-HIIT training program, including dynamic stretches and 3 minutes of cycling at 50 W. Following a 1-minute transition period, the official test began. The C-HIIT program, conducted using a cycle ergometer (ergoline 100 K, Germany), consisted of 10 60-second working intervals at 90% PPO, with each interval followed by a 60-second active recovery period at 25% PPO. Throughout the exercise, participants were instructed to maintain a pedal cadence between 60 and 65 rpm.

Before starting R-HIIT, participants performed approximately 10 minutes of warm-up, including dynamic stretches and squats without load. The R-HIIT protocol consisted of 10 60s working intervals, each separated by 60s recovery periods. During the working intervals, participants performed barbell back squats with a load of 20% bodyweight. To ensure proper form, participants placed the barbell on the trapezius and held it with both hands with the assistance of the researcher. They were instructed to squat down to approximately 90° knee flexion, maintaining contact between their feet and the floor, and were supposed to achieve full hip and knee extension at the end of each repetition. The eccentric phase of each squat lasted 1 second, while the concentric phase was performed as fast as possible to a standing position. The maximum number of squats completed per working interval was 30 reps. To control the tempo of the movements, a metronome set at 60 beats per minute was utilized. If participants were unable to complete the movement at the prescribed tempo, a brief rest in a standing position with the load was allowed. After each working interval, the researcher removed the barbell and repositioned it on the participant’s trapezius 5 seconds before the next exercise bout.

### Experimental measures

#### Anthropometric measurements

All anthropometric data are shown in Table 1. Participants’ height (cm) and body weight (kg) were measured using an ultrasonic tester (DHM-200, China). BMI was then calculated by dividing the subjects’ weight (kilograms) by the square of their height (meters). Additionally, body composition was assessed by dual X-ray absorptiometry (Lunar, United States).

#### Incremental exercise test

V̇O_2max_ is determined through a graded incremental exercise test (GXT) conducted on a cycling ergometer (ergoline 100 K, Germany). The test started with a 5-minute warm-up period at 50 W power. Subsequently, the workload was increased by 15 W every minute from 70 W until the participants reached voluntary exhaustion. Exhaustion was defined as the inability to maintain a speed of 60 revolutions/min for more than 5 seconds in succession despite verbal encouragement. During the GXT, expired gases were analyzed using a gas analyzer (AEI moxus, United States). Additionally, heart rate (HR) was continuously measured through the Polar RS400 to determine if participants reached their V̇O_2max_, they had to meet at least three out of the following four criteria: (1) a plateau of V̇O_2_ (i.e., change ≤2.1 mL/kg/min), (2) a final respiratory exchange ratio (RER) of ≥1.1, (3) maximum heart rate within 10 bpm of the age-predicted maximum [210-(0.65 × age)] [18, 24], and (4) a rating of perceived exertion (RPE) of ≥18. V̇O_2max_, calculated as the mean of the highest values obtained during three consecutive 10-second periods (30 seconds total), is presented in Table 1.

When the exercise intensity could not be maintained for the full 60 seconds, PPO was identified at the work rate coincident with volitional fatigue. PPO was calculated as W_final_ + (t/T)•W_inc_, W_final_ is the last power output (W) completed for 60 seconds, t (s) is the amount of time reached in the final uncompleted stage, T (s) is the duration of each stage (60 s), and Winc (W) is the workload increment (15 W).

#### Cardio-ankle vascular index and blood pressure text

Before warm-up, immediately and 30 min after both C-HIIT and R-HIIT, CAVI was measured using the VaSera VS-1500 vascular screening system (Fukuda Denshi, Beijing, China). Subjects were lying supine on a bed with all four limbs extended, and their information, such as name, gender, birth date, height, and weight was entered into the machine. The heart sound detector was placed near the sternum to monitor the heart sounds, the electrodes were clamped on the right and left wrists, and four cuffs were wrapped around the right and left upper arms and ankles [25]. When the first and second heart sounds were detected in the phonocardiogram, the START button was pressed. The average of the right and left CAVI (⊿CAVI) was calculated, and its change from baseline in the same trial was used for later analysis. The VaSera VS-1500 vascular screening system also measured blood pressure (BP).

#### Heart rate variability

HRV was measured simultaneously with ⊿CAVI. The assessment of all variables related to HRV was conducted at three different time points: (a) before warm-up, (b) immediately after exercise, and (c) 30 minutes. Cardiac autonomic modulation in HRV was measured using an electrocardiographic workstation (Shenzhen Boying Medical Instrument Technology Co, China) with subjects in the supine position and electrocardiogram electrodes placed on their wrists. The system obtained the values of every R-R interval independently, and 5 minutes were chosen for analysis. The Kubios software (v.3.5.0, HRV analysis, Finland) was used for HRV data processing. In the present analysis, the time domain variable of HRV utilized was the root mean square of successive differences between adjacent normal R-R intervals (RMSSD). The frequency domain variable was the high-frequency (HF) power of 0.15–0.4 Hz, as well as the LF/HF ratio. Among these parameters, the RMSSD and HF represent vagal activity, and LF/HF ratio is a measure of the cardiovascular autonomic balance. To address the skewness and violation of normality assumptions in the raw values of RMSSD, HF, a natural log (ln) transformation was applied (lnRMSSD, lnHF).

#### Blood sample collection and analysis

The whole blood (15 ml) was drawn from an antecubital vein by a skilled nurse previous, 5 minutes and 35 minutes after each exercise bout. After drawing the blood samples, the whole blood samples were allowed to clot at room temperature for 30 min, and then centrifuged at 3000 rpm for 10 min at 4 °C. Following centrifugation, the separated serum samples were frozen and stored at − 80 °C. The serum levels of cTnT and NT-proBNP were measured using ELISA kits (Albion, China). The kits had an intra-assay coefficient of variation of less than 10%. All analyses were conducted using an automatic microplate reader (E 601, Germany).

### Statistical analyses

Statistical analyses were performed using SPSS Statistics (version 26, IBM, United States). Data were presented as mean ± standard deviation (SD). Normality assumptions were checked using Shapiro-Wilk tests. A 2 × 3 two-way ANOVA with repeated measures was used to examine the changes in ⊿CAVI, SBP, lnHF, lnRMSSD, LF/HF ratio, cTnT and NT-proBNP across the two modes (C-HIIT and R-HIIT) observed points (Pre, 0-min post and 30-min point). *Post-hoc* analyses, using the Bonferroni’s correction factor were performed for cases in which the main effect was significant. Partial eta squared (*η*_*p*_^*2*^) was calculated to determine the magnitude of main and interaction effects and was categorized as small (0.01), medium (0.06), and large (0,14), respectively. Cohen’s d was used as a measure of effect size, with small, medium, and large effects equal to 0.2, 0.5, and 0.8, respectively. All analyses used an alpha level of *P* < 0.05 to indicate statistical significance, while a highly significant difference was indicated by *P* < 0.01.

## Results

Table 2 shows a significant time × group interaction effect (*P* = 0.019, *η*_*p*_^*2*^ = 0.182), and the time main effect was found in ⊿CAVI (*P* < 0.001, *η*_*p*_^*2*^ = 0.729). C-HIIT and R-HIIT were decreased in ⊿CAVI 0-min post (− 0.88 ± 0.43, − 1.53 ± 0.63, respectively) and 30-min post (− 0.72 ± 0.64, − 0.81 ± 0.78, respectively) compared to pre-exercise values. In addition, R-HIIT was reduced more significantly than C-HIIT immediately after exercise (− 0.60 ± 0.30, *P* = 0.043, *d* = 0.92).

**Table 2:** ⊿CAVI, SBP, HRV, cTn-T, NT-proBNP values before and after exercise

	C-HIIT	R-HIIT		*P*	
			Interaction effect	Group	Time
⊿CAVI					
Pre	6.24 ± 0.67	6.29 ± 0.72			
0-min Post	5.36 ± 0.64^*#^	4.76 ± 0.66^*#^	**0.019**	0.464	**< 0.001**
30-min Post	5.52 ± 0.74^#^	5.48 ± 0.78^#^			
SBP (mmHg)					
Pre	123 ± 10	124 ± 14			
0-min Post	129 ± 11^#^	126 ± 10^#^	0.179	0.580	**0.001**
30-min Post	124 ± 9	120 ± 9			
lnHF (ms)					
Pre	7.22 ± 0.97	7.11 ± 0.75			
0-min Post	4.59 ± 1.00^#^	4.01 ± 1.45^#^	0.519	0.313	**< 0.001**
30-min Post	6.41 ± 0.89^#^	5.94 ± 1.34^#^			
lnRMSSD (ms^2^)					
Pre	4.17 ± 0.52	4.07 ± 0.36			
0-min Post	2.86 ± 0.50^#^	2.56 ± 0.94^#^	0.746	0.341	**< 0.001**
30-min Post	3.70 ± 0.47^#^	3.53 ± 0.74^#^			
LF/HF					
Pre	0.88 ± 0.96	1.17 ± 1.03			
0-min Post	2.84 ± 2.37^#^	1.95 ± 1.52^#^	0.105	0.931	**0.001**
30-min Post	1.26 ± 1.09	1.73 ± 1.53			
cTnT(ng/L)					
Pre	1.10 ± 1.48	1.10 ± 1.48			
5-min Post	1.31 ± 1.84^#^	1.47 ± 1.92^#^	0.293	0.804	**0.023**
35-min Post	0.96 ± 1.04	1.28 ± 1.77			
NT-proBNP(ng/L)					
Pre	93.08 ± 41.63	111.75 ± 35.42			
5-min Post	134.07 ± 60.71^#^	129.27 ± 33.64^#^	0.208	0.789	**0.001**
35-min Post	109.97 ± 54.10	110.00 ± 30.35			

*P < 0.05 significantly different between C-HIIT and R-HIIT, ^#^*P* < 0.05 significantly different from previous; C-HIIT: cycling-type high intensity interval training; R-HIIT: resistance-type high intensity interval training; ⊿CAVI: the average of the right and left cardio-ankle vascular index; SBP: systolic blood pressure; lnHF: the frequency domain variable was the high-frequency; lnRMSSD: the root mean square of successive differences between adjacent normal R-R intervals; cTnT: cardiac troponin-T; NT-proBNP: amino-terminal pro-B-type natriuretic peptide

Systolic blood pressure (SBP) for two HIIT trials is presented in Table 2, with no significant time × group interaction effect (*P* = 0.179, *η*_*p*_^*2*^ = 0.083) and group main effect (*P* = 0.580, *η*_*p*_^*2*^ = 0.016) was observed for SBP. However, the time main effect was significant (*P* = 0.001, *η*_*p*_^*2*^ = 0.304). SBP decreased significantly from pre to 0-min post both in C-HIIT (5.55 ± 4.20) and R-HIIT (2.27 ± 7.91) and was not different from testing values 30-min post.

Table 2 shows changes in lnHF, lnRMSSD and LF/HF, no significant time × group interaction effect (*P* = 0.591, *η*_*p*_^*2*^ = 0.025; *P* = 0.746, *η*_*p*_^*2*^ = 0.012; *P* = 0.105, *η*_*p*_^*2*^ = 0.108, respectively) and group main effect (*P* = 0.313, *η*_*p*_^*2*^ = 0.051; *P* = 0.341, *η*_*p*_^*2*^ = 0.045; *P* = 0.931, *η*_*p*_^*2*^ = 0.000, respectively) was observed. But there was a significant time main effect for lnHF, lnRMSSD and LF/HF (*P* < 0.001, *η*_*p*_^*2*^ = 0.782; *P* < 0.001, *η*_*p*_^*2*^ = 0.693; *P* = 0.001, *η*_*p*_^*2*^ = 0.302, respectively). In the C-HIIT and R-HIIT trials, lnHF was significantly deceased 0-min post (− 2.63 ± 0.81, − 3.10 ± 1.58, respectively) and 30-min post (− 0.80 ± 0.88, − 1.16 ± 1.02, respectively), lnRMSSD was significantly decreased 0-min post (− 1.31 ± 0.39, − 1.51 ± 0.92, respectively) and 30-min post (− 0.47 ± 0.35, − 0.54 ± 0.68, respectively), LF/HF was elevated from pre to 0-min post (1.73 ± 1.53, 0.79 ± 1.09, respectively) and was not different from resting values 30-min post.

Table 2 shows changes in cTnT and NT-proBNP, no significant time × group interaction effect (*P* = 0.293, *η*_*p*_^*2*^ = 0.059; *P* = 0.208, *η*_*p*_^*2*^ = 0.077, respectively) and the group main effect (*P* = 0.804, *η*_*p*_^*2*^ = 0.003; *P* = 0.789, *η*_*p*_^*2*^ = 0.004, respectively) was observed. However, a significant time main effect was observed for cTnT and NT-proBNP (*P* = 0.023, *η*_*p*_^*2*^ = 0.193; *P* = 0.001, *η*_*p*_^*2*^ = 0.334, respectively). cTnT and NT-proBNP significantly increased from pre to 5-min post both in C-HIIT (0.21 ± 0.39, 40.98 ± 42.12) and R-HIIT (0.37 ± 0.50, 17.52 ± 36.13), and was not different from resting value 35-min post.

## Discussion

The major finding was that there were no significant differences in the acute effects on HRV, SBP, cTnT, and NT-proBNP between C-HIIT and R-HIIT. However, we found that R-HIIT was more effective than C-HIIT in reducing ⊿CAVI immediately after exercise. Additionally, the reduction in ⊿CAVI was observed to have sustained for 30 minutes in both C-HIIT and R-HIIT.

Our study observed a significant improvement in ⊿CAVI following both C-HIIT and R-HIIT, which was supported by a meta-analysis [10]. The mechanism underlying the reduction in arterial stiffness after acute exercise can be attributed to the increased blood flow and shear stress induced by exercise, which leads to an enhanced release of nitric oxide (NO) from endothelial cells [24]. The release of NO causes smooth muscle to relax in response to the constant pressure caused by the increased blood flow, resulting in a reduction in arterial stiffness. Additionally, our study observed a greater decrease in ⊿CAVI values immediately after completing R-HIIT compared to C-HIIT. This difference could be attributed to the distinct contraction patterns of the skeletal muscles. Specifically, the squat-based R-HIIT involves both concentric and eccentric contractions, while C-HIIT is predominantly characterized by concentric contractions. Previous research has shown that eccentric contractions induce greater cardiac output compared to concentric contractions [26], resulting in higher levels of NO release [27]. As a result, R-HIIT, which induces higher cardiac output, was more effective than C-HIIT in reducing arterial stiffness.

Our study observed an immediate elevation in SBP following both C-HIIT and R-HIIT. This can be attributed to the significant increase in skeletal muscle oxygen consumption during exercise, leading to an increased output per beat by the heart and subsequently higher blood flow to replenish the oxygen consumed by the muscles. As a result, the increased pressure on the blood vessel walls leads to an increase in SBP. This transient increase in SBP is only a stress response, and in our study, SBP returned to baseline 30 minutes after exercise, what was found in our study is consistent with previous research by Zheng [13], who reported SBP in healthy men elevated immediately after interval training and returned to baseline 40 minutes after the training. However, other studies have reported patients with hypertension and prehypertension experienced a decrease in SBP minutes or hours after acute exercise [28]. This phenomenon appears to be absent in healthy populations. Our study also found no difference in acute changes in SBP after C-HIIT and R-HIIT. This is similar to the study of Armas [29], which reported no significant differences in acute responses in SBP after a 7-minute bodyweight resistance exercise circuit and a 7-minute C-HIIT in healthy populations, indicating that exercise mode has no effect on SBP.

The analysis of HRV variables in time domains (lnRMSSD) revealed a reduction, while the frequency domains lnHF showed a reduction immediately after exercise. These findings suggest that sympathetic activity was dominant and parasympathetic regulation was suppressed. Increased sympathetic activity promotes improvement in cardiac function (autoregulation, cardiac output) in a short period of time in response to acute exercise stimulation [30]. The exercise-induced disruption of cardiac autonomic modulation is generally considered temporary, with the recovery time being related to exercise intensity [16]. Our study found that although markers of parasympathetic (lnRMSSD and lnHF) displayed a rising tendency 30 minutes after exercise, they were still significantly below the baseline. This indicates that the cardiac autonomic modulation system has not fully recovered at this time and that suggested additional training should not be undertaken at this time. Furthermore, our study found no significant difference between C-HIIT and R-HIIT in their effects on HRV. These results are consistent with a study conducted by Schaun [3], which compared the effects of whole-body R-HIIT and C-HIIT on HRV and concluded that the mode of HIIT did not affect the cardiac autonomic modulation system. Huang [31] compared continuous training with HIIT at matched identical exercise intensity and revealed no difference in HRV destruction and recovery after acute exercise. These findings suggest that exercise intensity rather than mode is the main factor influencing HRV changes. In our study, R-HIIT and C-HIIT were intensity-matched by RPE, so there was no difference in acute destruction and recovery in HRV after two HIIT protocols.

Our study found that cTnT was increased after both R-HIIT and C-HIIT. This increase in cTnT can be attributed to multiple factors. During acute exercise, the cardiomyocytes experience mechanical stress, leading to an overload of free radicals, elevated body temperature, and prolonged acidosis [31]. Consequently, the permeability of the cardiomyocyte membrane increases, resulting in the release of cTnT from the cytoplasm into the bloodstream [32]. Additionally, the production of reactive oxygen species after acute exercise, along with myocardial ischemia and hypoxia, can cause damage to the myocardial cell membrane, leading to a temporary rise in cTnT [33, 34]. Some studies have also suggested a possible association between the acute elevation of cTnT after acute exercise and skeletal muscle injury [35], although there is currently controversy surrounding this claim. Our study found that cTnT returned to baseline 35 minutes after exercise. However, some studies have reported that cTnT was elevated for 2–3 hours after exercise [31]. We did not observe in cTnT for a longer period after exercise, which is a limitation of our study. In addition, our study found no significant difference in cTnT acute changes induced between C-HIIT and R-HIIT. Nie [36] reported that HIIT and MICT at the same absolute intensity don’t have significant differences in changes in cTnT. In other words, exercise intensity is the main factor affecting cTnT changes after acute exercise. Exercise intensity was the same for C-HIIT and R-HIIT in our study.

Our study observed a significant acute increase in NT-proBNP levels after both C-HIIT and R-HIIT. In healthy populations, induced NT-proBNP elevation after exercise has been shown to have cytoprotective and growth-regulating effects, preventing myocardial fibrosis and inadequate hypertrophy [37]. However, it is worth noting that the NT-proBNP returned to baseline 35 minutes after both C-HIIT and R-HIIT in our study. This finding contradicts a meta-analysis that reported that NT-proBNP levels remained elevated compared to resting levels for 24 hours after endurance exercise in healthy individuals [38]. These results suggest that HIIT may induce less cardiac stress compared to endurance training. Furthermore, we found that there was no significant difference in the changes of NT-proBNP induced by these two modes of HIIT. García [39] reported that acute exercise-induced increases in NT-proBNP are primarily determined by the duration of exercise and the subjects’ training levels. Serrano [40] also reported that 21 healthy subjects performed continuous running at different durations and intensities. It was found that duration of exercise was the main factor affecting post-exercise NT-proBNP levels rather than exercise intensity. In our study, the duration for two HIIT protocols and subjects’ training levels was the same. This is different from cTnT, a finding supported by a meta-analysis by Papamichail [38].

Several limitations of the present study should be acknowledged. First, this is an acute exercise intervention design, and thus, the long-term effects of C-HIIT and R-HIIT on the cardiovascular system deserve attention. Second, the reference range of the findings is limited as the subjects in this study were healthy young men, indicating the need for further investigation in populations with cardiovascular disease. Last, our study only utilized the behind-the-neck squat in R-HIIT, potentially resulting in overexertion of the lower body. Therefore, future research should aim to improve the R-HIIT program.

## Conclusions

Our study demonstrated that R-HIIT was more effective than C-HIIT arterial in decreasing arterial stiffness and that C-HIIT and R-HIIT could induce the same acute effect on cardiac autonomic modulation. In addition, the increase in cardiac biomarkers (cTnT, NT-proBNP) caused by C-HIIT and R-HIIT was reversible.

### Supplementary Information

**Additional file 1.**

## Data Availability

The data underlying this paper, which includes the privacy of the individuals involved, cannot be made public for the following reasons. These data will be shared with the respective authors upon reasonable request. If you need the data, you can contact me via my e-mail: 497697348@qq.com.

## Abbreviations

HIIT: High-intensity interval training
C-HIIT: Cycling-type high intensity interval training
R-HIIT: Resistance-type high intensity interval training
CAVI: Cardio-ankle vascular index
HRV: Heart rate variability
SBP: Systolic blood pressure
cTnT: Cardiac troponin-T
NT-proBNP: Amino-terminal pro-B-type natriuretic peptide
HR_peak_: Peak heart rate
MICT: Moderate-intensity continuous training
RPE: Rating of perceived exertion
RMSSD: The root mean square of successive differences between adjacent normal R-R intervals
HF: The frequency domain variable was the high-frequency
HR: Heart rate
HR_max_: Maximal heart rate
V̇O_2max_: Maximal oxygen consumption
PPO: Peak power output
NO: Nitric oxide
